# Tb^3+^ and Bi^3+^ Co-Doping of Lead-Free Cs_2_NaInCl_6_ Double Perovskite Nanocrystals for Tailoring Optical Properties

**DOI:** 10.3390/nano13030549

**Published:** 2023-01-29

**Authors:** Yang Yu, Wei Zhou, Cheng Li, Peigeng Han, Hui Li, Kun Zhao

**Affiliations:** 1 Institute of Ultrafast Optical Physics, MIIT Key Laboratory of Semiconductor Microstructure and Quantum Sensing & Department of Applied Physics, Nanjing University of Science and Technology, Nanjing 210094, China; 2 State Key Laboratory of Molecular Reaction Dynamics, Dalian Institute of Chemical Physics, Chinese Academy of Sciences, Dalian 116023, China; 3 Institute of Molecular Sciences and Engineering, Institute of Frontier and Interdisciplinary Science, Shandong University, Qingdao 266237, China

**Keywords:** lead-free double perovskite, lanthanide ion doping, Cs_2_NaInCl_6_, nanocrystals

## Abstract

Lead halide perovskites have achieved remarkable success in various photovoltaic and optoelectronic applications, especially solar cells and light-emitting diodes (LEDs). Despite the significant advances of lead halide perovskites, lead toxicity and insufficient stability limit their commercialization. Lead-free double perovskites (DPs) are potential materials to address these issues because of their non-toxicity and high stability. By doping DP nanocrystals (NCs) with lanthanide ions (Ln^3+^), it is possible to make them more stable and impart their optical properties. In this work, a variable temperature hot injection method is used to synthesize lead-free Tb^3+^-doped Cs_2_NaInCl_6_ DP NCs, which exhibit a major narrow green photoluminescence (PL) peak at 544 nm derived from the transition of Tb^3+ 5^D_4_→^7^F_5_. With further Bi^3+^ co-doping, the Tb^3+^-Bi^3+^-co-doped Cs_2_NaInCl_6_ DP NCs are not only directly excited at 280 nm but are also excited at 310 nm and 342 nm. The latter have a higher PL intensity because partial Tb^3+^ ions are excited through more efficient energy transfer channels from the Bi^3+^ to the Tb^3+^ ions. The investigation of the underlying mechanism between the intrinsic emission of Cs_2_NaInCl_6_ NCs and the narrow green PL caused by lanthanide ion doping in this paper will facilitate the development of lead-free halide perovskite NCs.

## 1. Introduction

Lead (Pb) halide perovskites have received great research attention because of their remarkable performance in photovoltaic and optoelectronic applications, including light-emitting diodes (LEDs), solar cells, and optical pumping lasers [1,2,3,4,5,6,7]. Despite their promising properties, lead halide perovskites have not been commercialized due to their intrinsic instability and lead toxicity [8,9]. The water solubility of lead halide perovskites is associated with lead toxicity diseases involving the nervous system. To address the instability and toxicity of lead halide perovskites, researchers have been actively pursuing the development of lead-free perovskite alternatives. Sn^2+^ and Ge^2+^ have been used to replace Pb^2+^ to synthesize lead-free halide perovskites [10,11]. However, the Sn^2+^ and Ge^2+^ cations tend to oxidize to Sn^4+^ and Ge^4+^ in the ambient environment. Double perovskites (DPs) as lead-free perovskite variants containing one monovalent B^+^ cation and one trivalent B^3+^ cation to generate the [BX_6_]^5−^ and [B’X_6_]^3−^ octahedra, resulting in a three-dimensional (3D) structure of A_2_BB’X_6_ (A = Rb, Cs; B = Na, Ag; B’ = Bi, In, Sc, Er; X = I, Br, Cl), have received tremendous research attention due to their intense photoluminescence (PL), non-toxicity, and high stability. Nevertheless, most kinds of air-stable DP nanocrystals (NCs) exhibit forbidden optical transitions or wide band gaps [12,13,14,15], making their optoelectronic applications impractical and pushing researchers to improve their optical and optoelectronic properties.

Lanthanide ion (Ln^3+^) incorporation is a viable method to enhance the stability of DP NCs and impart optical properties via B’-site replacement. Several examples of Ln^3+^ ion doping in DP NCs have been proven experimentally, such as Ho^3+^ ion doping into Cs_2_AgNaInCl_6_ [16], Yb^3+^ and Mn^2+^ ions being doped into Cs_2_AgBiX_6_ [17], Yb^3+^ and Er^3+^ ions being doped into Cs_2_AgInCl_6_ [18], and Tb^3+^ and Sb^3+^ ions being doped into Cs_2_NaInCl_6_ [19,20]. Ln^3+^ commonly generates unique emissions with a narrow bandwidth as compared to transition metal ions, whose emissions are rather broader [21]. The energy transfer between lanthanide ions can be utilized to modulate the emissions in Ln^3+^-doped luminescent materials. In Ln^3+^-doped DPs, the energy transfer channel from the perovskite host or self-trapped excitons (STEs) to Ln^3+^ ions has been confirmed [16,22,23].

Lead-free Cs_2_NaInCl_6_ DP NCs were synthesized using a variable temperature hot injection method in this study. The undoped Cs_2_NaInCl_6_ NCs had little PL, while the Tb^3+^-doped Cs_2_NaInCl_6_ DP NCs exhibited a characteristic emission of Tb^3+^ from the transitions of ^5^D_4_→^7^F_5_, ^5^D_4_→^7^F_6_, ^5^D_4_→^7^F_4_, and ^5^D_4_→^7^F_3_. With further Bi^3+^ co-doping, the Tb^3+^-Bi^3+^-co-doped Cs_2_NaInCl_6_ NCs were not only directly excited at 280 nm but were also excited at 310 nm and 342 nm, with the latter obtaining a higher PL intensity because partial Tb^3+^ ions in Tb^3+^-Bi^3+^-co-doped NCs are excited by more efficient energy transfer from the Bi^3+^ to the Tb^3+^ ions. This work investigates the underlying mechanism between the intrinsic emission of Cs_2_NaInCl_6_ DP NCs and the narrow green PL resulting from Ln^3+^ ion doping. It will facilitate the development of lead-free halide perovskite NCs and expand their application in optoelectronics.

Due to its high stability and nontoxicity, lead-free Cs_2_NaInCl_6_ DP NC has attracted excellent research attention, especially on its optical properties and optoelectronic applications. However, more efforts are needed to achieve tunable band gaps and light emission in specific applications. Other lanthanide ion doping, such as Ho^3+^, Er^3+^, and Yb^3+^ [16,17,18], can be used to modulate the band gap and light emission. The mixing of monovalent or trivalent metals in DP NCs, that is, isovalent doping, such as Na^+^/Ag^+^, Ag^+^/Cu^+^, and In^3+^/Sb^3+^ [23,24,25,26,27], is an effective strategy. On the other hand, heterovalent doping has also made great progress in stages, among which divalent manganese ion is one of the representative dopants [28,29]. Although these doping strategies provide a variety of options for band gap and light emission modulation, the strategies are prone to defect formation, so the optimization of nanomaterial growth and device fabrication is critical for optoelectronic applications. In addition, the dimensional regulation of DP NCs could help to adjust their electronic structures to extend the absorption spectra from the ultraviolet-visible region to the near-infrared region, which would widen their applications in photovoltaic devices [30].

## 2. Materials and Methods

Materials: Indium acetate (In(OAc)_3_, 99.99%), silver acetate (Ag(OAc), 99.99%), oleic acid (OA, 90%), and 1-octadecene (90%) were purchased from Alfa Aesar Chemical Co., Ltd. (Shanghai, China). Terbium acetate hydrate (Tb(OAc)_3_∙nH_2_O, 99.99%), sodium acetate (Na(OAc), 99.99%), cesium acetate (Cs(OAc), 99.99%), oleylamine (OLA, 80%), and n-hexane (97%) were purchased from Shanghai Aladdin Biochemical Technology Co., Ltd. (Shanghai, China). Chlorotrimethylsilane (TMSCl, 99%) and bismuth acetate (Bi(OAc)_3_, 99.99%) were purchased from Sigma-Aldrich Co., Ltd. (Shanghai, China). All chemicals were utilized directly as they were received without further purification.

Sample Preparation: An optimized hot injection method was used to synthesize the Cs_2_NaInCl_6_ NCs. In detail, 131.4 mg In(OAc)_3_, 36.9 mg Na(OAc), and 125.0 mg Cs(OAc) were mixed with octadecene (10 mL), oleylamine (0.65 mL), and oleic acid (2.9 mL), placed in a 50 mL two-necked flask, and heated at 105 °C for 80 min under vacuum. Using nitrogen protection, the reaction solution was heated at a rate of 7 °C/min to 190 °C, with 0.5 mL of TMSCl being quickly injected at 180 °C, after 20 s, and quickly cooled down to room temperature in an ice-water bath. After that, the mixture was centrifuged at 10,000 rpm for 20 min. The supernatant was separated off. To obtain colloidal Cs_2_NaInCl_6_ NCs, the precipitate was washed with 5 mL of toluene, centrifuged for 5 min at 10,000 rpm, redispersed with sonication in 5 mL of hexane, and centrifuged for 5 min at 5000 rpm. The Tb^3+^- and Bi^3+^-ion-doped Cs_2_NaInCl_6_ NCs were synthesized using the same method, except for adding varied feed ratios of Tb(OAc)_3_∙nH_2_O or Bi(OAc)_3_ at the first step.

Characterization: Powder X-ray diffraction (PXRD) was carried out at room temperature with a PANalytical Empyrean diffractometer (Malvern Panalytical Ltd., Malvern, UK) under Cu K radiation (λ = 1.54056). The transmission electron microscopy (TEM) measurements and energy dispersive spectroscopy (EDS) mapping were conducted using the JEM-2100 (Japan Electronics Co., Ltd., Tokyco, Japan) for microstructure observation and elemental distribution analysis. PerkinElmer 8300 (Perkin Elmer, Waltham, MA, USA)was used for inductively coupled plasma optical emission spectrometer (ICP-OES) measurements to determine the concentration of specified elements in the samples. Optical diffuse reflectance was measured using a Shimadzu UV 2550 spectrometer (Shimadzu, Kyoto, Japan) equipped with an integrating sphere over the spectral range from 200 nm to 900 nm, with BaSO_4_ as the complete reflectance reference. The absorption spectra were obtained by transforming the reflectance data using the Kubelka–Munk equation, $F\left(R\right)={\left(1-R\right)}^{2}{\left(2R\right)}^{2}=\alpha /S$, where *R* is the reflectance, and *α* and *S* are the absorption and scattering coefficients, respectively. Photoluminescence excitation (PLE) and PL spectra were obtained using the Horiba PTI QuantaMaster 400 (Horiba, Shanghai, China). The PL lifetime measurement was carried out using a home-built time-correlated single photon counting system. The excitation beam was a nanosecond pulse diode laser, and the optical detector was a single photon counting module.

## 3. Results and Discussion

**Figure 1:**
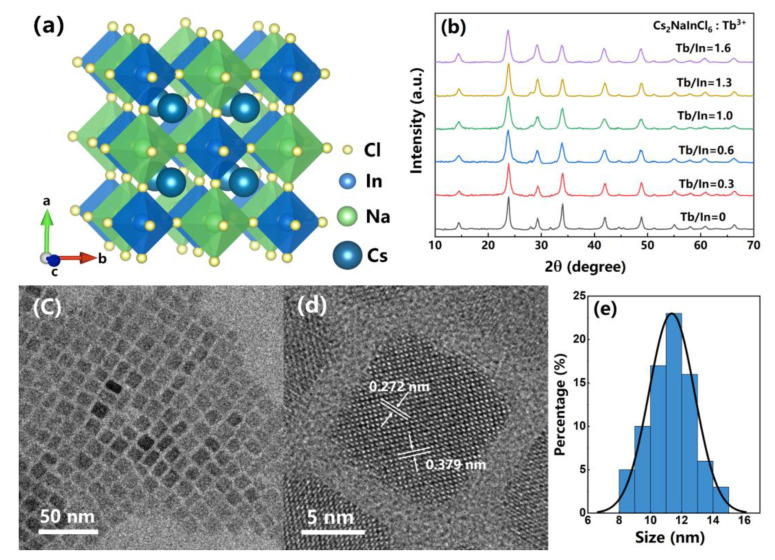
(**a**) Cs_2_NaInCl_6_ NCs lattice structure, with yellow, blue, and green spheres representing Cl, In, and Na atoms, and bigger blue spheres representing Cs atoms. (**b**) PXRD patterns of the undoped and Tb^3+^-doped Cs_2_NaInCl_6_ DP NCs. (**c**) TEM and (**d**) HRTEM images of Tb^3+^-doped Cs_2_NaInCl_6_ DP NCs. (**e**) Histogram of the size distribution of Tb^3+^-doped Cs_2_NaInCl_6_ NCs.

As shown in Figure 1a, the lattice structure of the Cs_2_NaInCl_6_ DP NCs crystallizes in a highly symmetric cubic structure (F$\overset{-}{m}$3m space group). Corner-connected [NaCl₆]^5−^ and [InCl₆]^3−^ octahedrons construct a 3D framework with Cs^+^ inserted in the octahedron’s cavities [31,32]. Figure 1b shows the PXRD patterns of the undoped and Tb^3+^-doped Cs_2_NaInCl_6_ DP NCs. The XRD peaks of the undoped Cs_2_NaInCl_6_ NCs at 2θ values of 14.4°, 23.8°, 28.1°, 29.3°, 34.0°, 41.9°, 48.9°, and 55.0° correspond to (111), (220), (311), (222), (400), (422), (440), and (620) lattice planes, respectively [33,34]. There is no detectable impurity phase in the doped NCs, implying that no phase separation occurred and the lattice structure remains unchanged. The actual doping concentrations in these samples are far lower than the feeding ratios revealed by the ICP-OES measurements (Table 1). Tb^3+^ ions are considered to replace In^3+^ ions in the crystalline lattice of Cs_2_NaInCl_6_ DP NCs [34,35,36]. The EDS result indicates that the molar ratio of major elements in Cs_2_NaInCl_6_ NCs is close to the ratio of 2:1:1:6 (Table 2). As shown in Figure 1c, the TEM image shows that the cubic-shaped Tb^3+^-doped Cs_2_NaInCl_6_ DP NCs are evenly distributed with an average size of about 11 nm. The high-resolution TEM (HRTEM) picture of Tb^3+^-doped NCs demonstrates excellent crystallinity with 0.272 nm and 0.379 nm lattice spacing values matched to the (400) and (220) crystal planes (Figure 1d). 

**Figure 2:**
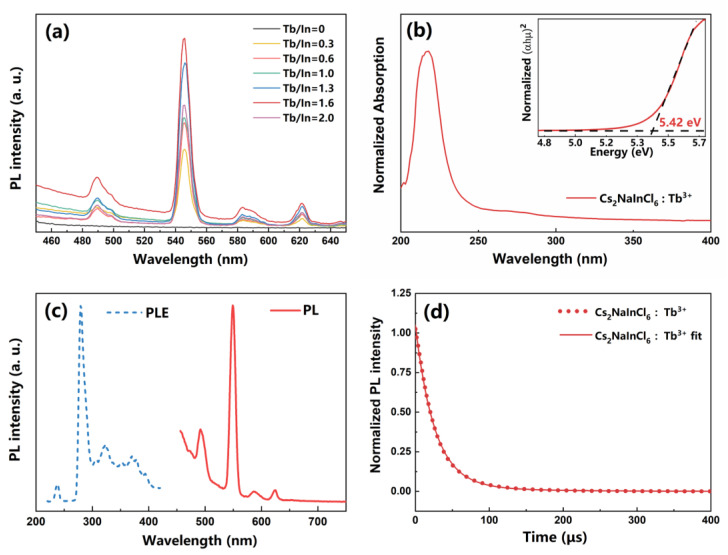
(**a**) PL spectra under 280 nm excitation for Cs_2_NaInCl_6_ NCs with different Tb^3+^ doping ratios. (**b**) Diffuse reflection absorption spectrum; insert shows the corresponding Tauc plots of Tb^3+^-doped Cs_2_NaInCl_6_ NCs. (**c**) PLE and PL, (**d**) time-resolved PL spectra of the Tb^3+^-doped Cs_2_NaInCl_6_ NCs with fitting curves.

The optical properties of the Tb^3+^-doped Cs_2_NaInCl_6_ DP NCs were investigated using steady-state PL and absorption spectra. The PL spectra under 280 nm excitation for Cs_2_NaInCl_6_ NCs with different Tb^3+^ doping ratios are shown in Figure 2a. The Tb^3+^-doped Cs_2_NaInCl_6_ NCs exhibit a major narrow green PL peak at 544 nm derived from the transition of Tb^3+ 5^D_4_→^7^F_5_, with three other small emission peaks at 490 nm, 583 nm, and 622 nm derived from the transitions of Tb^3+ 5^D_4_→^7^F_6_, ^5^D_4_→^7^F_4_, and ^5^D_4_→^7^F_3_ [21,37]. The PL intensity increases dramatically when the feeding ratio of doping agents is increased, while the peak location stays constant. The optimum Tb/In atomic feeding ratio is 1.6. After further increasing the Tb^3+^ doping amount, the PL intensity drops due to the concentration quenching effect. For clarity, the following discussion will focus on the optimal doping ratio samples. In the diffuse reflection absorption spectra (Figure 2b), a major absorption peak at 217 nm is observed for the Tb^3+^-doped NCs. The corresponding Tauc plot exhibits a wide band gap of 5.42 eV. The PLE and PL spectra of the Tb^3+^-doped NCs are shown in Figure 2c. A major green emission peak at 550 nm with a large Stokes shift of 270 nm is observed. The narrow green emission should be attributed to the characteristic emission of Tb^3+^ [21,37], while the undoped NCs are non-luminous, which indicates that Tb^3+^ ions are excited via energy transfer channels from the Cs_2_NaInCl_6_ host to Tb^3+^ ions. The photophysical properties of the Tb^3+^-doped NCs were investigated using transient PL spectra. As shown in Figure 2d, the PL lifetime of the Tb^3+^-doped NCs is fitted with exponential function with an extremely long lifetime (τ = 62 μs), which is attributed to the recombination process of excited Tb^3+^ ions involving an energy transfer from the Cs_2_NaInCl_6_ host to excite Tb^3+^ ions.

**Figure 3:**
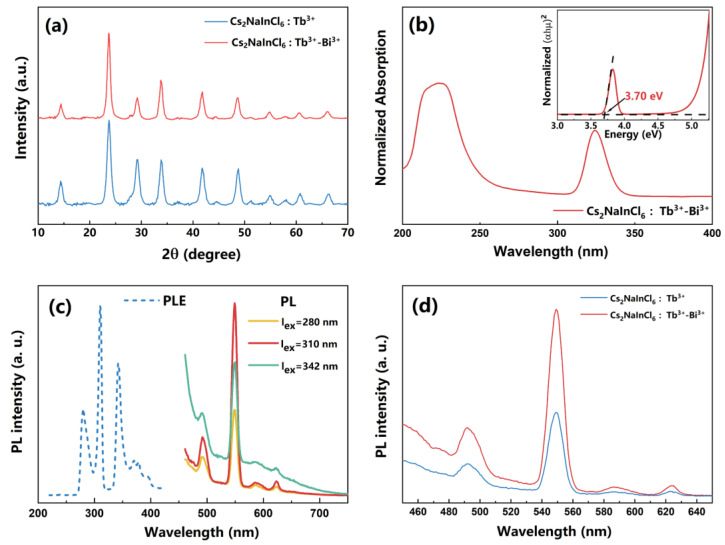
(**a**) PXRD patterns of the Tb^3+^-singly-doped and Tb^3+^-Bi^3+^-co-doped Cs_2_NaInCl_6_ NCs. (**b**) Diffuse reflection absorption spectrum; insert shows the corresponding Tauc plots of the Tb^3+^-Bi^3+^-co-doped Cs_2_NaInCl_6_ NCs. (**c**) PLE and PL spectra of the Tb^3+^-Bi^3+^-co-doped Cs_2_NaInCl_6_ NCs. (**d**) PL spectra of the Tb^3+^-singly-doped and Tb^3+^-Bi^3+^-co-doped Cs_2_NaInCl_6_ NCs.

**Figure 4:**
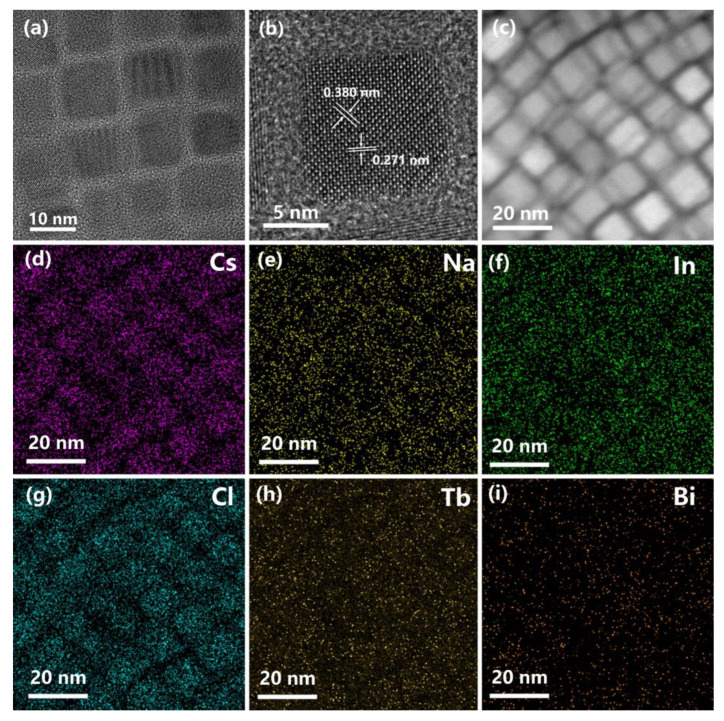
(**a**) TEM, (**b**) HRTEM, (**c**) high-angle annular dark field (HAADF) images of the Tb^3+^-Bi^3+^-co-doped Cs_2_NaInCl_6_ NCs. (**d**–**i**) EDS elemental mappings of Cs, Na, In, Cl, Tb, and Bi localized in the Tb^3+^-Bi^3+^-co-doped Cs_2_NaInCl_6_ NCs.

The use of a co-doping strategy to modify the PL properties of metal halide DPs has attracted a great deal of attention [23,24,25,26]. The trivalent Bi^3+^ cations are chosen for co-doping with Tb^3+^ ions for Cs_2_NaInCl_6_ DP NCs. The PXRD patterns of the Tb^3+^-Bi^3+^-co-doped Cs_2_NaInCl_6_ NCs are shown in Figure 3a. The XRD patterns of Tb^3+^-Bi^3+^-co-doped NCs are similar to those of Tb^3+^-singly-doped NCs, indicating that the lattice structure is not significantly altered with an additional 5% Bi^3+^ dopant. The TEM image of the Tb^3+^-Bi^3+^-co-doped NCs is shown in Figure 4a. The HRTEM image of the Tb^3+^-Bi^3+^-co-doped NCs shows excellent crystallinity with lattice spacing values of 0.271 nm and 0.380 nm matching to the (400) and (220) crystal planes (Figure 4b), indicating that the trace Bi^3+^ dopant does not significantly change the lattice distance. The mixing of Bi^3+^ and In^3+^ trivalent ions is considered to be random in the B’-site in the crystalline lattice [31]. The EDS element mappings show that the Cs, Na, In, Cl, Tb, and Bi elements are homogeneously distributed in the NCs, indicating the character of a single-phase compound (Figure 4d–i). 

The absorption spectra of the Tb^3+^-Bi^3+^-co-doped NCs are shown in Figure 3b. Except for the major absorption peak at 223 nm, another absorption peak is detected at 325 nm, which should be attributed to the correlated 6s^2^-6s^1^6p^1^ transitions of Bi^3+^ ions. The insert Tauc plot shows a band gap of 3.70 eV for the Tb^3+^-Bi^3+^-co-doped NCs, indicating that Bi^3+^ co-doping can lower the band gap of the DP NCs because the energy can transfer directly from the Bi^3+^ ions to the Tb^3+^ ions. As shown in Figure 3c, the PLE spectrum of Tb^3+^-Bi^3+^-co-doped NCs shows three PLE peaks at 280 nm, 310 nm, and 342 nm, which is different from the Tb^3+^-singly-doped NCs. Similar PL emission peaks at 549 nm with large Stokes shifts of 269 nm, 238 nm, and 206 nm are observed. With Bi^3+^ co-doping, which decreased the energy of absorbed photons, the samples are not only directly excited at 280 nm but are also excited at 310 nm and 342 nm. The latter obtain a higher PL intensity because partial Tb^3+^ ions in co-doped NCs are excited through more efficient energy transfer channels from the Bi^3+^ to the Tb^3+^ ions, consistent with the previous reports [16,22]. The PL spectra of the Tb+-singly-doped and Tb^3+^-Bi^3+^-co-doped Cs_2_NaInCl_6_ NCs are shown in Figure 3d. The higher PL intensity of Tb^3+^-Bi^3+^-co-doped NCs than that of Tb^3+^-singly-doped NCs demonstrates that the energy transfer channel built by introducing Bi^3+^ ions is more favorable for the highly efficient luminescence of Tb^3+^ ions than the intrinsic excitation band.

## 4. Conclusions

In conclusion, we report the Tb^3+^ and Bi^3+^ doping of Cs_2_NaInCl_6_ DP NCs for narrow green PL. The Tb^3+^-doped Cs_2_NaInCl_6_ DP NCs exhibit a major narrow green PL peak at 544 nm, derived from the transition of Tb^3+ 5^D_4_→^7^F_5_, with three other small emission peaks at 490 nm, 583 nm, and 622 nm derived from the transitions of Tb^3+ 5^D_4_→^7^F_6_, ^5^D_4_→^7^F_4_, and ^5^D_4_→^7^F_3_. The ultra-long PL lifetime of about 62 μs corresponds to the recombination process of excited Tb^3+^ ions, involving an energy transfer from the Cs_2_NaInCl_6_ host to excite Tb^3+^ ions. The additional two stronger PLE peaks at 310 nm and 342 nm caused by further Bi^3+^ co-doping indicate that partial Tb^3+^ ions in Tb^3+^-Bi^3+^-co-doped NCs are not only directly excited at 280 nm but are also more efficiently excited through energy transfer channels from the Bi^3+^ to the Tb^3+^ ions. The emission intensity of Tb^3+^-Bi^3+^-co-doped NCs is much higher than that of Tb^3+^-singly-doped NCs, indicating that introducing Bi^3+^ ions is more favorable for the highly efficient luminescence of Tb^3+^ ions by providing more efficient energy transfer channels. This work provides an effective method for producing lead-free halide DPs with excellent optical properties, and this mechanism has great potential for tailoring the optical properties of DPs.

## Figures and Tables

**Table 1:** The ICP-OES results of different feed ratios of Tb^3+^-doped Cs_2_NaInCl_6_ DP NCs.

Cs_2_NaInCl_6_:Tb^3+^ Feeding Ratios	In (mg/L)	Tb (mg/L)	In (%)	Tb (%)	Tb/In Actual Ratios
Tb/In = 0.3	17.68	0.035	15.37	0.022	0.001432
Tb/In = 0.6	13.22	0.049	11.50	0.031	0.002681
Tb/In = 1.0	17.33	0.106	15.07	0.067	0.004424
Tb/In = 1.3	13.63	0.097	11.85	0.061	0.005147
Tb/In = 1.6	16.67	0.121	14.50	0.076	0.005250
Tb/In = 2.0	34.63	0.285	30.11	0.179	0.005952

**Table 2:** The EDS results of Tb^3+^-doped Cs_2_NaInCl_6_ DP NCs.

Element	Weight%	Atomic%
Na	3.83	10.19
Cl	34.93	60.18
In	20.99	11.16
Cs	39.95	18.36
Tb	0.29	0.11

## Data Availability

The data presented in this study are available upon request from the corresponding author.
